# Investigating the effects of maltreatment and acute stress on the concordance of blood and DNA methylation methods of estimating immune cell proportions

**DOI:** 10.1186/s13148-023-01437-5

**Published:** 2023-02-28

**Authors:** Abner T. Apsley, Laura Etzel, Waylon J. Hastings, Christine C. Heim, Jennie G. Noll, Kieran J. O’Donnell, Hannah M. C. Schreier, Chad E. Shenk, Qiaofeng Ye, Idan Shalev

**Affiliations:** 1 Department of Biobehavioral Health, The Pennsylvania State University, 219 Biobehavioral Health Building, University Park, PA 16802 USA; 2 Department of Molecular, Cellular, and Integrated Biosciences, The Pennsylvania State University, University Park, PA USA; 3 Corporate Member of Freie Universität Berlin, and Humboldt-Universität Zu Berlin, Berlin Institute of Health (BIH), Institute of Medical Psychology, Charité – Universitätsmedizin Berlin, Berlin, Germany; 4 Department of Human Development and Family Studies, The Pennsylvania State University, University Park, PA USA; 5 Yale Child Study Center, Yale School of Medicine, Yale University, New Haven, CT USA; 6 Department of Obstetrics Gynecology and Reproductive Sciences, Yale School of Medicine, Yale University, New Haven, CT USA; 7 Department of Pediatrics, The Pennsylvania State University College of Medicine, Hershey, PA USA

**Keywords:** Immune cell proportions, DNA methylation, Complete blood count, Childhood maltreatment, Acute psychosocial stress

## Abstract

**Background:**

Immune cell proportions can be used to detect pathophysiological states and are also critical covariates in genomic analyses. The complete blood count (CBC) is the most common method of immune cell proportion estimation, but immune cell proportions can also be estimated using whole-genome DNA methylation (DNAm). Although the concordance of CBC and DNAm estimations has been validated in various adult and clinical populations, less is known about the concordance of existing estimators among stress-exposed individuals. As early life adversity and acute psychosocial stress have both been associated with unique DNAm alterations, the concordance of CBC and DNAm immune cell proportion needs to be validated in various states of stress.

**Results:**

We report the correlation and concordance between CBC and DNAm estimates of immune cell proportions using the Illumina EPIC DNAm array within two unique studies: Study 1, a high-risk pediatric cohort of children oversampled for exposure to maltreatment (*N* = 365, age 8 to 14 years), and Study 2, a sample of young adults who have participated in an acute laboratory stressor with four pre- and post-stress measurements (*N* = 28, number of observations = 100). Comparing CBC and DNAm proportions across both studies, estimates of neutrophils (*r* = 0.948, *p* < 0.001), lymphocytes (*r* = 0.916, *p* < 0.001), and eosinophils (*r* = 0.933, *p* < 0.001) were highly correlated, while monocyte estimates were moderately correlated (*r* = 0.766, *p* < 0.001) and basophil estimates were weakly correlated (*r* = 0.189, *p* < 0.001). In Study 1, we observed significant deviations in raw values between the two approaches for some immune cell subtypes; however, the observed differences were not significantly predicted by exposure to child maltreatment. In Study 2, while significant changes in immune cell proportions were observed in response to acute psychosocial stress for both CBC and DNAm estimates, the observed changes were similar for both approaches.

**Conclusions:**

Although significant differences in immune cell proportion estimates between CBC and DNAm exist, as well as stress-induced changes in immune cell proportions, neither child maltreatment nor acute psychosocial stress alters the concordance of CBC and DNAm estimation methods. These results suggest that the agreement between CBC and DNAm estimators of immune cell proportions is robust to exposure to child maltreatment and acute psychosocial stress.

**Supplementary Information:**

The online version contains supplementary material available at 10.1186/s13148-023-01437-5.

## Background

Blood proportions of immune cells, such as monocytes, lymphocytes, and granulocytes, are tightly regulated in healthy individuals [1]. Deviations from normative immune cell proportions can be used to predict and monitor the progression of many diseases and conditions including chronic inflammation, viral infections, arterial disease, coronary heart disease, gastroenteritis, endocrine disorders, leukemia, and lymphoma [2–6]. Thus, it is critical to accurately measure the proportion of circulating immune cell subtypes for both diagnosis and monitoring of disease progress [7, 8].

Beyond clinical implications, it is critical to accurately measure immune cell proportions for basic research investigating connections between environmental/psychosocial exposures and health. Molecular profiles of whole blood, such as gene expression (9), DNA methylation (DNAm) [10], and telomere length [11], are often derived by averaging the measurement of these molecular features across a heterogeneous mixture of immune cell subtypes within each blood sample. However, each immune cell subtype has different functions and molecular signatures (e.g., methylation patterns, gene expression, telomere length distribution), and thus, the resulting molecular profiles of whole blood are dependent upon the immune cell proportions of the sample [10, 12]. The variability of immune cell proportions—whether due to stress or other factors—across biological samples has implications for the accuracy and reliability of genomic studies. For example, controlling for immune cell proportions in epigenome-wide association studies (EWAS) can lead to improved biological interpretability, whereas not accounting for immune cell proportions in EWAS can lead to false positive results [13–15].

The complete blood count (CBC) is the most widely used clinical test for determining immune cell proportions [6, 16] and analyzes major immune cell components via flow cytometry sorting. Despite its ubiquitous application in clinical practice, the CBC method has some disadvantages in population research settings. The application of flow cytometry to determine CBC estimates requires the collection of blood which is often difficult to obtain, especially in pediatric cohorts. Additionally, CBC tests must be performed within 24 h of sample collection and cannot be performed on stored (e.g., dried or frozen) samples. In contrast to CBC estimators, molecular profiles can be reliably generated from dried or frozen blood samples, as well as from noninvasive saliva or buccal swab samples. These alternative molecular methods for detecting immune cell proportions are of considerable interest for a broad range of research fields.

One alternative method for estimating immune cell proportions is the use of whole-genome DNAm data. Because of the unique pattern of methylation inherent in each cell type, methylation signals at specific 5'-cytosine-phosphate-guanine-3' (CpG) sites can be used to estimate immune cell proportions [17–19]. Houseman and colleagues developed the first statistical algorithm for estimating immune cell proportions using CpG probes from the Illumina 27 k DNAm array [20, 21], which was later updated by constructing reference sets for the Illumina 450 k DNAm array [14, 22–25]. Salas and colleagues provided an updated algorithm and a reference set specifically tailored to the Illumina EPIC DNAm array [26–28]. Each iteration of immune cell estimators was designed to make estimations with greater accuracy than previous versions. More recently, Salas and colleagues released a revised reference set for the Illumina EPIC DNAm array enabling estimation of a much larger variety of immune cell subtypes than previously possible [29]. Although the accuracy of some of these reference sets and algorithms has been validated in various clinical populations (e.g., patients with head and neck cancer, ovarian cancer, down syndrome, and obesity) [20], in cord blood [30], and in older adults [14], to our knowledge, no work has been done to determine the effects of exposure to child maltreatment (CM), as well as acute psychosocial stress, on the concordance of CBC and DNAm estimates of immune cell proportions.

Exposure to early life adversity (ELA), such as CM, has been shown to alter the DNAm profiles of individual genes such as *NR3C1, BDNF, PRF1, SLC6A4, OXTR, COMT*, *AVP*, and *CRF* [31–37]*,* as well as alter the DNAm profiles of immune cells [38–41]. Moreover, the effects of CM and other adverse environmental exposures on DNAm profiles could extend beyond candidate genes and introduce DNAm alterations on a genome-wide scale [42, 43]. These alterations could influence the concordance of CBC and DNAm estimations of immune cell proportions if such alterations overlap with the CpG sites of established DNAm algorithms used to discriminate between immune cell subtypes. Therefore, it is important to validate the concordance of CBC and DNAm methods for estimating immune cell proportions in individuals who have experienced CM.

Beyond CM, changes in DNAm profiles in response to acute psychosocial stress could also alter the concordance of CBC and DNAm methods for estimating immune cell proportions. Evidence suggests that immune cell DNAm levels within promoter regions of stress-related genes respond to acute psychosocial stress [44, 45]. It is unknown whether these alterations persist after the acute stressor has subsided, and whether or not additional areas of the methylome are impacted by acute stress. In addition, DNAm levels in rats and mice are reported to be sensitive to acute stress in various brain regions such as the hippocampus, cortex, and periaqueductal gray area [46, 47]. Given the observed correlations between brain and blood cell methylomes [48, 49], it is plausible that similar changes in DNAm levels of immune cells could be occurring. Changes in DNAm profiles of immune cells in response to acute psychosocial stress can have implications for study designs in which biological samples are collected before, during, and following stressful events.

This study aims to assess the concordance of CBC and DNAm methods for estimating immune cell proportions in blood when implemented 1) cross-sectionally in children who have been investigated for CM, and 2) in four repeated samples of young adults undergoing an acute psychosocial stressor. We hypothesized that CM and acute psychosocial stress would alter the concordance between CBC and DNAm methods for estimating immune cell proportions, with lower concordance under conditions of stress. To test our hypotheses, we compared CBC and DNAm estimates of immune cell proportions in two studies. In Study 1, we included children from the ongoing Child Health Study (CHS), a large multidisciplinary study designed to provide prospective, longitudinal data on the health and development of children with and without a history of investigations for CM (physical and sexual abuse, and neglect). Study 1 was used to determine the effects of CM on the concordance of CBC and DNAm methods for estimating immune cell proportions. In Study 2, we used a sample of healthy young adults exposed to the Trier Social Stress Test (TSST), a laboratory stressor shown to promote robust sympathetic and adrenal stress responses [50] and elicit changes in immune cell proportions within individuals across time [51]. Study 2 was used to determine the effects of acute psychosocial stress on the concordance between CBC and DNAm methods of estimating immune cell proportions. We used the Illumina EPIC DNAm method of estimating immune cell proportions [29] and provide supplementary analyses using the previous Illumina EPIC DNAm iteration [26–28].

## Results

### Study 1: Testing the impact of child maltreatment on the concordance between CBC and DNAm estimates of immune cell proportions

Study 1 was designed such that it included both individuals with a history of CM investigations (for sexual abuse, physical abuse, or neglect) who were oversampled, and non-exposed individuals without a history of CM investigations, resulting in a total *N* = 365 (*N* = 307 youth with a maltreatment history and *N* = 58 comparison youth). Tests for demographic differences by CM status demonstrated that the CM group had, older age (*p* = 0.035), higher BMI (*p* = 0.002), more advanced pubertal stage (*p* < 0.001), and lower household income (*p* < 0.001) (see Table 1 for complete demographic comparisons).

**Table 1:** Study 1 Descriptive statistics

	Comparison (*N* = 58)	CM History (*N* = 307)	Total (*N* = 365)	*p* value
	Mean (SD)/Count (%)			
Sex				
Male	28 (48.3%)	158 (51.5%)	186 (51.0%)	0.762
Female	30 (51.7%)	149 (48.5%)	179 (49.0%)	
Age (years)	11.11 (1.4)	11.53 (1.4)	11.47 (1.4)	**0.035**
BMI	19.93 (4.9)	22.27 (5.9)	21.89 (5.8)	**0.002**
Puberty Status	2.08 (0.9)	2.58 (1.0)	2.50 (1.0)	** < 0.001**
Income $10,000/year	5.74 (3.8)	3.37 (3.1)	3.75 (3.3)	** < 0.001**
Race				
White	45 (77.6%)	210 (68.4%)	255 (69.9%)	0.214
Not white	13 (22.4%)	97 (31.6%)	110 (30.1%)	
Ethnicity				
Hispanic	3 (5.2%)	48 (15.6%)	51 (14.0%)	0.057
Non-Hispanic	55 (94.8%)	259 (84.4%)	314 (86.0%)	
CBC proportions				
Neutrophils	48.97 (9.6)	49.07 (9.9)	49.05 (9.8)	0.941
Lymphocytes	38.75 (8.8)	38.56 (9.0)	38.59 (9.0)	0.879
Monocytes	7.57 (2.1)	7.57 (1.9)	7.57 (1.9)	0.985
Eosinophils	4.13 (3.3)	4.16 (3.7)	4.16 (3.6)	0.936
Basophils	0.54 (0.4)	0.55 (0.4)	0.55 (0.4)	0.863
DNAm proportions				
Neutrophils	47.45 (10.9)	47.55 (10.2)	47.53 (10.3)	0.948
Lymphocytes	38.43 (9.9)	38.29 (9.3)	38.31 (9.3)	0.920
Monocytes	7.63 (2.1)	7.64 (1.8)	7.64 (1.9)	0.979
Eosinophils	4.16 (3.6)	4.09 (4.2)	4.10 (4.1)	0.899
Basophils	0.14 (0.4)	0.15 (0.3)	0.15 (0.4)	0.935

Bold entries indicate a significance level of *p* < 0.05

*p* values are given as a result of *t* tests or Chi-square tests between control and maltreatment groups

**Fig. 1:**
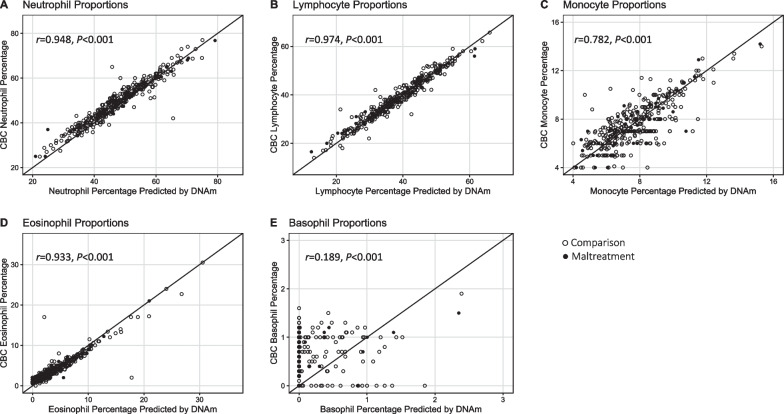
Study 1 CBC estimates of immune cell proportions are displayed on the y-axis and DNAm estimates are displayed on the x-axis. A 45-degree reference line is shown to display the trajectory of perfect concordance between CBC and DNAm estimates

There were no statistically significant differences in mean values of CBC or DNAm estimates of all immune cell subtypes when comparing CM and comparison groups (Table 1). Across all participants, CBC and DNAm estimates of immune cell proportions were highly correlated with one another for each cell type, with the exception of basophils (Pearson’s correlation: neutrophils: *r* = 0.948, *p* < 0.001; lymphocytes: *r* = 0.974, *p* < 0.001; monocytes: *r* = 0.782, *p* < 0.001; eosinophils: *r* = 0.933, *p* < 0.001; basophils: *r* = 0.189, *p* < 0.001; Additional file 2: Table S1). Excluding zero observations of basophils for CBC and DNAm estimates increased the correlation for basophils (*r* = 0.380*, p* < 0.001). Figure 1 shows CBC estimates plotted as a function of DNAm estimates with a 45-degree identity reference line and stratified by CM status. Distributions of CBC and DNAm estimates for each cell type are shown in Additional file 1: Figure S1.

**Fig. 2:**
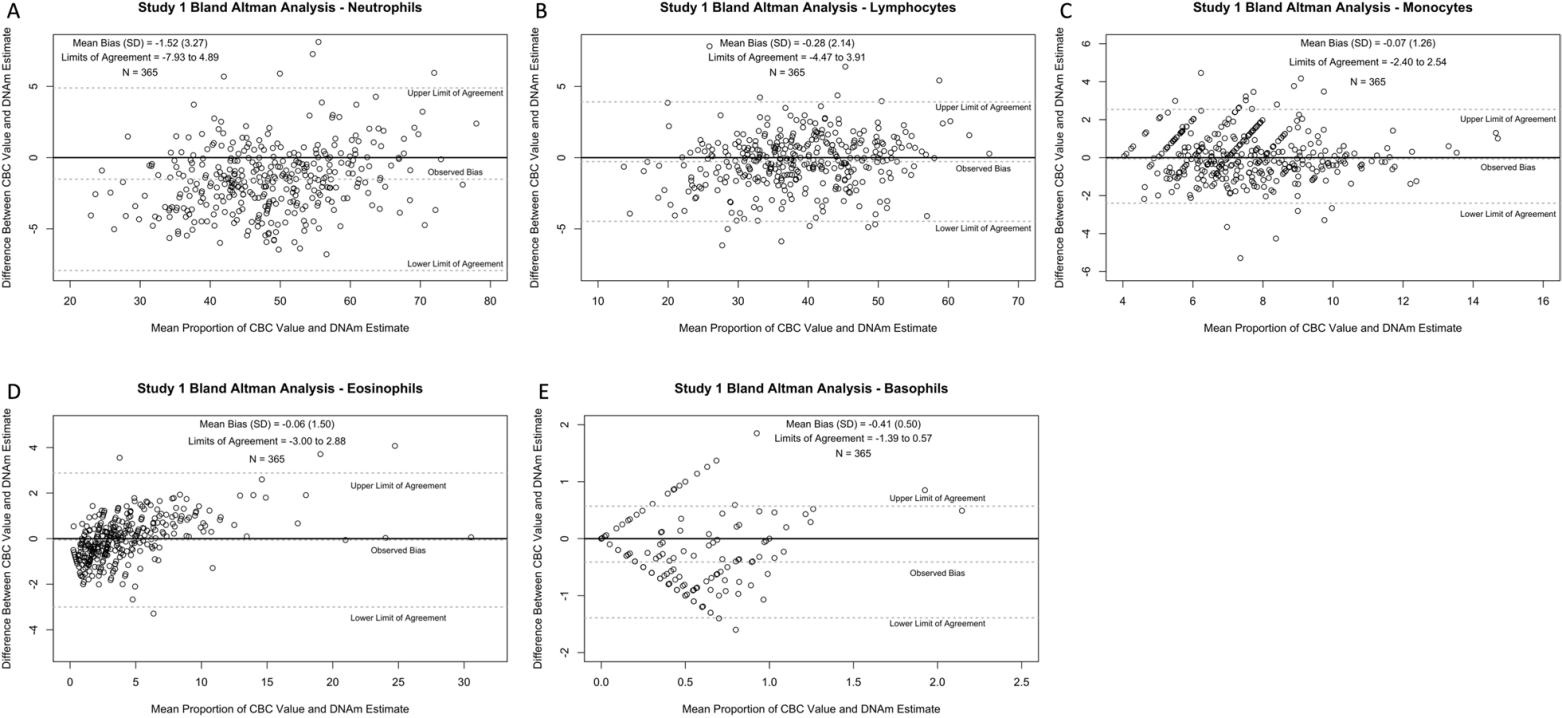
Study 1: Bland–Altman plots of neutrophils, lymphocytes, monocytes, eosinophils, and basophils. Differences (y-axis) are defined as [DNAm estimates − CBC estimates]. Positive observed biases indicate DNAm estimates overestimating CBC estimates, and negative observed biases indicate DNAm estimates underestimating CBC estimates

We next examined the agreement between CBC and DNAm estimates among each cell type using the Bland–Altman method. Neutrophil CBC and DNAm estimates were significantly different from one another (*β* = -1.52, *p* < 0.001) with DNAm estimates being, on average, 1.52% lower than CBC estimates (Fig. 2A). Lymphocyte CBC and DNAm estimates were also significantly different from one another (*β* = -0.28, *p* = 0.014), with DNAm estimates, on average, being 0.28% lower than CBC estimates (Fig. 2B). In contrast, monocyte and eosinophil CBC and DNAm estimates did not show significant differences from one another (monocytes: *β* = 0.07, *p* = 0.28; eosinophils: *β* = -0.06, *p* = 0.46; Fig. 2C, D). Basophil CBC and DNAm estimates were significantly different from one another (*β* = -0.41, *p* < 0.001), with DNAm estimates being, on average, 0.41% lower than CBC estimates (Fig. 2E). The data dispersion for all immune cell subtypes was varied, with neutrophils having the largest range (20%-80%) in observations and basophils having the smallest range (0%-2.5%). Additionally, all cell types had a similar dispersion of differences between CBC and DNAm estimates other than basophils, which had the greatest number of observations outside the upper and lower limits of agreement.

Immune cell proportion difference scores [DNAm estimate − CBC estimate] were regressed onto CM status and covariates (Table 2). Although lymphocytes showed a significant difference in the Bland–Altman analysis, only neutrophils (*β* = -1.77, *p* < 0.001) and basophils (*β* = -0.37, *p* < 0.001) showed significant intercepts in our regression analysis, meaning that only these two cell types had significant differences between DNAm and CBC estimates after accounting for covariates. CM status was not a significant predictor of differences in CBC and DNAm estimates for all immune cell subtypes (neutrophils: *β* = -0.02, *p* = 0.96; lymphocytes: *β* = 0.06, *p* = 0.85; monocytes: *β* = -0.14, *p* = 0.43; eosinophils: *β* = 0.04, *p* = 0.85; basophils: *β* = -0.06, *p* = 0.43; Table 2).

**Table 2:** Study 1 Multivariate linear regression results

	Neutrophils		Lymphocytes		Monocytes		Eosinophils		Basophils	
	Estimate	SE	Estimate	SE	Estimate	SE	Estimate	SE	Estimate	SE
Intercept	− 1.770***	**0.50**	− 0.222	0.32	0.323	0.18	− 0.121	0.23	− 0.369***	**0.07**
CM history	− 0.024	0.49	0.062	0.32	− 0.142	0.18	0.042	0.22	− 0.058	0.07
Sex	0.276	0.35	− 0.301	0.21	− 0.151	0.12	− 0.050	0.15	− 0.014	0.05
Age	0.147	0.16	− 0.028	0.10	− 0.124*	**0.06**	0.009	0.07	− 0.077**	**0.02**
Income	0.029	0.06	− 0.017	0.04	− 0.014	0.02	0.001	0.03	− 0.009	0.01
BMI	0.006	0.03	0.018	0.02	0.004	0.01	− 0.040**	**0.01**	0.005	0.00
Pubertal stage	0.177	0.23	− 0.207	0.15	0.202*	**0.08**	− 0.080	0.10	0.105**	**0.03**
Race	− 0.067	0.40	− 0.009	0.26	0.549***	**0.15**	− 0.207	0.18	0.122*	**0.06**
Ethnicity	− 0.716	0.51	0.296	0.33	0.068	0.19	0.037	0.23	− 0.101	0.07

Models were constructed with difference scores (DNAm estimate − CBC estimate) as the outcome, CM as the predictor, and all demographic variables as covariates

Bold entries indicate a significance level of *p* < 0.05

* *p* < 0.05. ** *p* < 0.01. *** *p* < 0.001

### Study 2: Testing the impact of acute psychosocial stress on the concordance between CBC and DNAm estimates of immune cell proportions

Study 2 participants ranged in age from 18–24 years (mean age = 21.3, SD = 1.3), had an average BMI of 24.2 (SD = 3.7), and were primarily female (64.3%). This study was designed to test the impact of ELA, and thus approximately half of the study participants recruited reported exposure to multiple adverse events in early life (*N* = 13 ELA exposure young adults and *N* = 15 control young adults). Four repeated blood samples were collected before and after an acute laboratory stressor (see Methods). Since study 2 involved DNAm estimates generated using DNA extracted from peripheral blood mononuclear cells (PBMCs), we conducted comparisons only between estimates of lymphocytes and monocytes. Full demographic statistics for Study 2, including CBC and DNAm estimates for each time-point, are shown in Table 3.

**Table 3:** Study 2 Descriptive statistics

Demographic statistics		Immune cell proportion statistics				
	Total (*N* = 28)		Time 1 (*N* = 27)	Time 2 (*N* = 26)	Time 3 (*N* = 24)	Time 4 (*N* = 23)
	Mean (SD)/Count (%)		Mean (SD)	Mean (SD)	Mean (SD)	Mean (SD)
Sex		CBC proportions				
Male	10 (35.7%)	Monocytes	22.88 (5.7)	22.74 (6.4)	22.69 (6.1)	19.52 (4.5)
Female	18 (64.3%)	Lymphocytes	77.12 (5.7)	77.26 (6.4)	77.31 (6.1)	80.48 (4.5)
Age (years)	21.3 (1.3)	DNAm proportions				
BMI	24.2 (3.7)	Monocytes	23.72 (6.0)	22.91 (6.0)	22.62 (7.2)	20.98 (4.7)
Race		Lymphocytes	76.28 (6.0)	76.28 (6.0)	77.38 (7.2)	79.02 (4.7)
White	19 (67.9%)					
Not white	9 (32.1%)					
Group status						
ELA	13 (46.4%)					
Controls	15 (53.6%)					

**Fig. 3:**
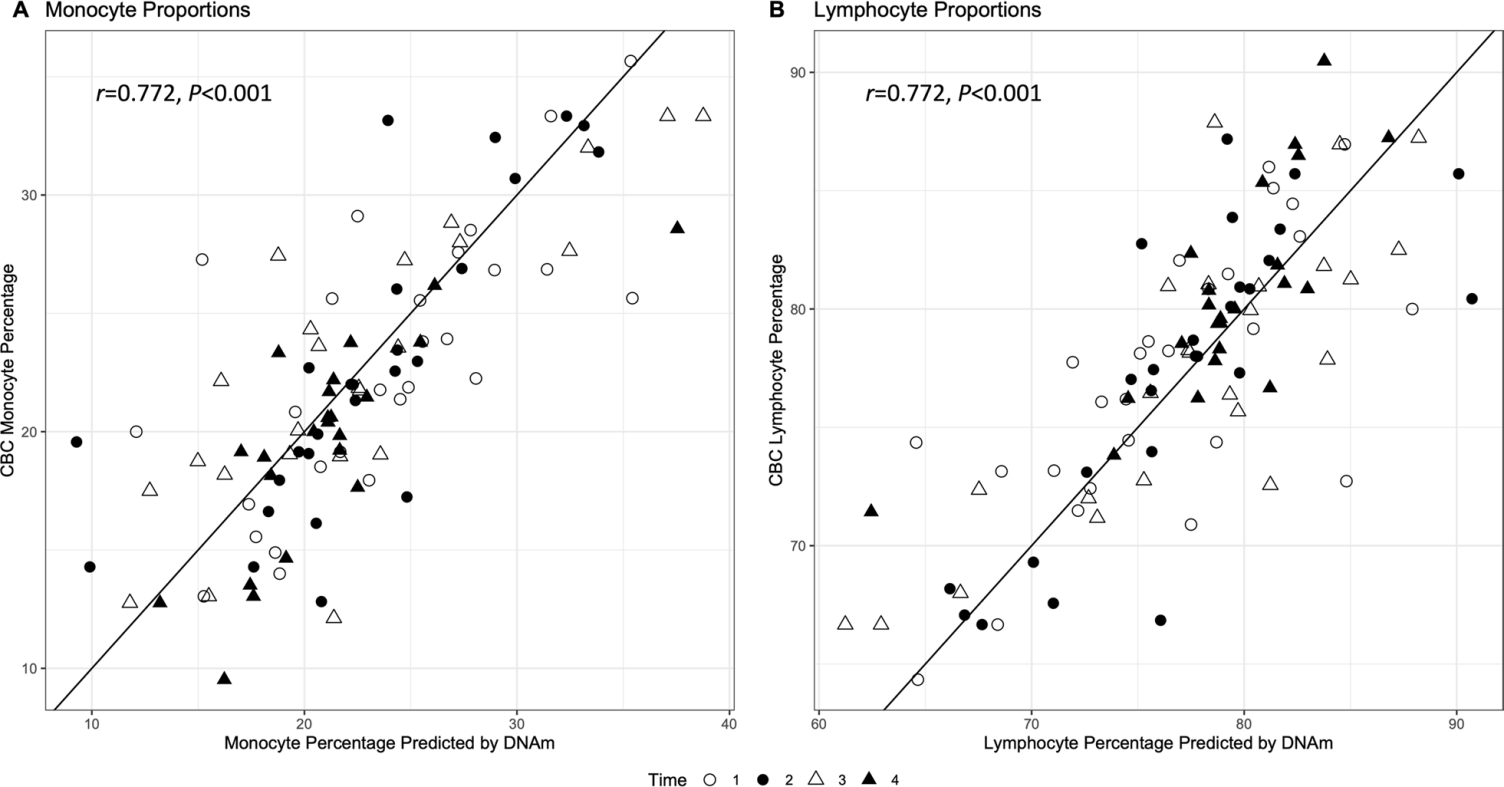
Study 2: CBC estimates of immune cell proportions are displayed on the y-axis and DNAm estimates are displayed on the x-axis. A 45-degree reference line is shown to display the trajectory of perfect concordance between CBC and DNAm estimates. See Additional file 2: Table S2 for statistical tests of differences between time-point concordances

Across all time-points and individuals, both monocytes and lymphocytes had a Pearson correlation coefficient of 0.78 (*p* < 0.001). When compared to time 1 (i.e., 30 min pre-stress), time 4 (i.e., 240 min post-stress) had significantly lower CBC (*β* = -3.24, *p* < 0.001) and DNAm estimates (*β* = -2.22, *p* = 0.02) for monocyte proportions. Additionally, compared to time 1, time 4 had significantly higher CBC (*β* = 3.24, *p* < 0.001) and DNAm estimates of lymphocyte proportions (*β* = 2.22, *p* = 0.02), highlighting significant changes in PBMC immune cell proportions in response to acute stress for both CBC and DNAm estimates. Figure 3 shows CBC estimates plotted as a function of DNAm estimates with a 45-degree identity reference line and stratified by time-point. Distributions of CBC and DNAm estimates for each cell type are shown in Additional file 1: Figure S2.

**Fig. 4:**
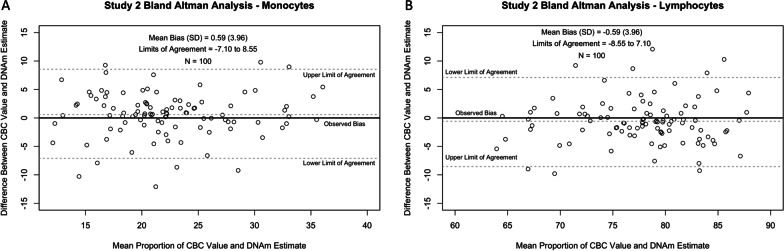
Study 2: Bland–Altman plots of monocytes and lymphocytes. Differences (y-axis) are defined as [DNAm estimates − CBC estimates]. Positive observed biases indicate DNAm estimates overestimating CBC estimates, and negative observed biases indicate DNAm estimates underestimating CBC estimates

We next examined the concordance between CBC and DNAm estimates for monocytes and lymphocytes using a repeated-measures Bland–Altman method. Monocyte CBC and DNAm estimates were not significantly different from one another (*β* = 0.59, *p* = 0.14) (Fig. 4A). Lymphocyte CBC and DNAm estimates were also not significantly different from one another (*β* = -0.59, *p* = 0.14) (Fig. 4B).

When regressing immune cell proportion difference scores [DNAm estimate − CBC estimate] onto time-point and covariates, time-point (i.e., pre- and post-stress) was not a significant predictor of difference scores for lymphocytes or monocytes (see Additional file 2: Table S2 for post hoc pairwise comparisons of time-points). Further, neither of the predicted intercepts for difference scores in monocytes and lymphocytes were significantly different from zero (monocytes: *β* = 1.16, *p* = 0.14; lymphocytes: *β* = -1.16, *p* = 0.14), indicating that there were no significant differences in DNAm and CBC estimate measurements across time-points. Additionally, ELA status was not a significant predictor of differences between DNAm and CBC estimates of immune cell proportions (monocytes: *β* = 2.52, *p* = 0.09; lymphocytes *β* = -2.52, *p* = 0.09), indicating that ELA status was not a confounding variable on the concordance between DNAm and CBC measurements over time (see Table 4 for full multilevel regression results).

**Table 4:** Study 2 multilevel modeling results

Fixed effects: monocytes (lymphocytes)		
	Estimate	SE
Intercept	1.156 (− 1.156)	0.78
Sex	0.351 (− 0.351)	1.14
Age	− 1.113* (1.113*)	**0.46**
BMI	0.313 (− 0.313)	0.19
Ethnicity	2.474* (− 2.474*)	**1.14**
Early life adversity	2.519 (− 2.519)	1.40
Time 2	− 0.474 (0.474)	0.94
Time 3	− 0.381 (0.381)	0.96
Time 4	0.936 (− 0.936)	0.97

Random effects		
Residual variance	11.52	
Individual-level variance	2.63	

Immune cell difference scores for monocytes and lymphocytes were modeled as a function of acute stress time-point with sex, age, BMI, ethnicity, and early life adversity status as covariates. Monocyte estimates were reported normally and lymphocyte estimates are reported in parentheses. Random effects for individuals were included for the intercept only

Bold entries indicate a significance level of *p* < 0.05

* *p* < 0.05

## Discussion

This study assessed the concordance between CBC and DNAm estimates of immune cell proportions using the recent Illumina EPIC DNAm method [29] in the context of CM and acute psychosocial stress. In Study 1, DNAm estimates were significantly correlated with CBC values for all immune cells. The lower correlation coefficients in monocyte and basophil estimates may be due to the limited range of these cell type proportions in comparison with neutrophils, lymphocytes, and eosinophils [52]. Using Bland–Altman analysis, DNAm estimates of both neutrophils and basophils were significantly lower than CBC estimates, though CM did not significantly explain any variation between estimation methods. DNAm estimates of lymphocytes, monocytes, and eosinophils showed no significant deviations from CBC estimates. In Study 2, DNAm estimates were significantly correlated with CBC values for both lymphocytes and monocytes. Additionally, using Bland–Altman analysis, DNAm and CBC estimates were not significantly different from one another for both monocytes and lymphocytes. While the acute stress manipulation caused significant changes in both monocyte and lymphocyte proportions over time across both approaches, it did not explain any variation in difference scores between CBC and DNAm estimates. Furthermore, in an exploratory analysis, ELA status did not impact the concordance of CBC and DNAm estimates for either lymphocytes or monocytes over time.

Previous studies investigating the concordance between DNAm and CBC estimates of immune cell proportions have been conducted in limited populations (e.g., patients with cancer, Down syndrome, obesity) [20] or niche tissues such as cord blood. Houseman and colleagues reported median difference scores between CBC and DNAm estimates to be 6.5% for neutrophils and 1.1% for monocytes when using their initial DNAm estimation library. Lymphocyte subset values ranged from 0.1–2.1% [20]. Importantly, the directionality of these results cannot be interpreted because the errors were reported as absolute values. Using the Houseman et al. DNAm estimation libraries, Koestler and colleagues reported the correlations of CBC and DNAm estimates to be around 0.60 for both lymphocytes and monocytes in PBMC samples [53], which is in line with our findings. Further, work by Accomando and colleagues [21] reported correlations between all immune cell subtypes when using the Houseman et al. DNAm estimation libraries, similar to our findings. However, Bland–Altman plots from this study exhibited negligible mean bias in immune cell subtype proportions.

Although prior work exists validating the original Houseman reference library, little research has been done to validate the concordance of CBC and DNAm estimates of immune cell proportions using the first EPIC array library generated by Salas and colleagues [26]. Salas and colleagues reported all lymphocyte cell subsets having DNAm estimates higher (> 1.25%) than CBC estimates. Monocyte values showed a similar trend with the median difference being less than 1% and neutrophils showed an opposite trend, with DNAm estimates being around 1.5% lower than CBC estimates. Each of these values are comparable to and in the same direction as the differences seen in Study 1 results when using the first EPIC array library for DNAm estimates, with the exception of neutrophils, which had a similar effect size but in the opposite direction (see Additional file 2: Tables S3–S7 for results of our analysis using the first EPIC array library generated by Salas and colleagues [26]).

To the best of the authors’ knowledge, there have been no previous studies assessing the concordance of CBC and DNAm estimates of immune cell proportions in a sample of maltreated children or in adults within the context of acute psychosocial stress. Further, this is the first study, to our knowledge, validating the recently released “FlowSorted.BloodExtended.EPIC” library [29] (see Methods). Study 1 provided a unique sample for testing the impact of CM on the concordance between CBC and DNAm estimates of immune cell proportions in a pediatric cohort. The experimental design employed by Study 2 allowed us to probe stress-induced temporal effects on the concordance of CBC and DNAm estimates of immune cell proportions. Additionally, repeated measurements of immune cell proportions in Study 2 were obtained over a 5 h time-window in response to an acute laboratory stressor, enabling us to detect the presence of temporal effects [54] of acute psychosocial stress on the concordance of immune cell proportion estimates. Contrary to expectations, we did not observe significant effects of CM or acute psychosocial stress on the concordance of CBC and DNAm immune cell estimates.

This study is not without limitations. For both studies, accurately comparing CBC and DNAm estimates of immune cell proportions is contingent on the assumption that whole blood immune cell proportions are equivalent in different blood tubes collected from the same venipuncture of an individual. Although most studies on the reliability of CBC estimates operate under the same premise [55–58], it is nevertheless still an assumption. In addition, all experiences of CM were grouped into one category which does not acknowledge the heterogeneity of biological effects resulting from differing age of first CM incident, type of CM, and severity of CM [59]. Future studies should examine the impact of detailed CM classification on the concordance of CBC and DNAm estimates. A limitation of Study 2 was the small sample size, larger studies with sufficient power are needed to more fully identify differences in immune cell estimates in response to acute psychosocial stress. Another limitation from Study 2 was the assumption that all individuals experienced a comparable increase in psychosocial stress from baseline to follow-up time-points during the acute stress procedure. Additionally, our exploratory analysis of the impact of ELA on CBC and DNAm estimate difference scores could have been improved by using a larger sample of ELA and control individuals.

The null effects of CM and acute psychosocial stress on the concordance of CBC and DNAm estimates of immune cell proportions may be due in part to minimal overlap between CpG sites altered by exposure to stress and the libraries of CpG sites used in DNAm estimators. Alternatively, alterations in DNAm patterns induced by CM or acute psychosocial stress at CpG sites overlapping with sites used in DNAm estimators of immune cell proportions may not be substantial enough to alter these estimates in a significant way. Future studies should explore whether specific CpG sites used in DNAm estimations of immune cell proportions overlap with other common CpG sites associated with physical or mental disorders. Additionally, future work should also be performed to test potential DNAm changes that take place in response to acute psychosocial stress over an extended period of time. Finally, we also recommend that more clinically practical methods [8, 60] of estimating immune cell proportions be tested for their accuracy in populations of individuals that have experienced CM and in individuals that have experienced acute psychosocial stress.

## Conclusions

Although significant differences in raw value estimation between CBC and DNAm exist for some immune cell proportions, CM and acute psychosocial stress did not alter the existing concordance of CBC and DNAm estimations of immune cell proportions. These findings extend previous research and suggest that study designs which include individuals with an exposure to adverse events, as well as study designs employing acute stress paradigms can rely on existing DNAm methods to estimate proportions of immune cell subtypes.

## Methods

### Study 1: Participants and procedures

Study 1 participants were members of the CHS [61], a large multidisciplinary study designed to provide prospective, longitudinal data on the health and development of children with and without a history of maltreatment. The CHS is currently recruiting a large state-wide cohort of children recently investigated for CM and non-maltreated comparison children. The goals of the CHS are to elucidate the multiple etiological processes, as well as mediators and moderators, believed to play a role in the onset and maintenance of adverse health outcomes among survivors of CM, and to better inform intervention opportunities to reverse the negative consequences of CM.

Recruitment for the CHS is ongoing. Children with a recent (< 12 months) report of CM exposure are identified in collaboration with Pennsylvania’s Statewide Child Welfare Information System (CWIS). Subjects with recent involvement in the CWIS are invited to participate in the study through home mailings and phone contact by study coordinators. Eligibility criteria include: 1) aged 8 to 13 years, 2) subject of a CWIS maltreatment report (i.e., an allegation is made and investigated) and agreement for participation within 12 months of CWIS involvement, and 3) agreement of participation by a non-abusing caregiver. Non-maltreated comparison children are recruited via targeted advertisements in the same Pennsylvania counties as children with a history of CM investigations. Eligibility for participation includes: 1) no previous CWIS reports or contact, and 2) demographic similarity to a maltreatment participant. After recruitment, participating families are invited to visit the Center for Healthy Children at The Pennsylvania State University for a full day of assessments and biospecimen collection. Approval from The Pennsylvania State University Institutional Review Board was granted, and informed assent (child) or consent (caregiver) was obtained from all participants.

Families of participants arrived at the Center for Healthy Children at 7:30 a.m. After an introduction to the study, youth underwent a physical exam followed by fasting whole blood collection in 10 mL and 4 mL EDTA tubes via antecubital venipuncture by a trained phlebotomist. Blood samples collected in 4 mL EDTA tubes were sent to Quest Diagnostics for CBC analyses within 24 h of collection. Genomic DNA was extracted from whole blood using a semi-automated approach (QIASymphony, Qiagen). Whole-genome DNAm levels were analyzed using the Illumina Infinium EPIC array.

Demographic information for each participant was collected by survey. Body mass index (BMI) was obtained via a trained staff member collecting weight and height information from each participant and then calculating their standard weight to height-squared ratio. Pubertal stage was assessed by a trained staff member by taking an average of self-reported Tanner pubertal status measures of breast growth and pubic hair growth [62]. Total household income before taxes was self-reported by caregivers using a 0–11 Likert scale (0 = Under $10,000/year, 1 = $10,000-$19,999/year… 11 = More than $120,000/year). Race was caregiver-reported and collapsed into a binary variable where “White” was coded as zero and all other races were coded as one. This was done to ensure a large enough sample size in each category to detect statistically significant effects of race. Ethnicity was caregiver-reported as either “Hispanic” or “Non-Hispanic”.

Cross-sectional data reported in Study 1 were drawn from the baseline (i.e., Time 1) assessment of currently enrolled CHS participants. Although recruitment for this cohort study is ongoing with a target enrollment of 700 children, an initial subset of 439 participants were available for the purpose of these analyses. Of the 439 participants who had completed Time 1 assessments, 435 consented to anthropometric measurements and 401 consented to and successfully completed blood draws (1 caregiver refusal, 33 participant refusals, 4 attempted but incomplete). The first batch for DNAm analysis constituted 286 total samples and 275 samples survived DNAm QC measures (see DNAm Quality Control and Immune Cell Proportion Estimations subsection). A second batch, including 126 samples, was submitted for DNAm analysis, of which all samples survived DNAm QC measures. Both batches combined amounted to a total of 401 individuals. Of these 401 samples, 36 were excluded from the current analyses due to failed/missing CBC tests or other missing covariates, making our final analytic sample a total of 365 participants. No demographic differences were present between groups included and excluded from the analysis, with the exception of BMI and pubertal status (BMI being higher in the included group and pubertal status being lower; *p* < 0.05).

### Study 2: Participants and procedures

Study 2 was comprised of a sample of 28 healthy individuals aged 18 to 25 years. Participants were recruited by word of mouth and advertisements on campus bulletin boards. During a visit to The Pennsylvania State University’s Clinical Research Center, participants were subjected to the Trier Social Stress Test (TSST) followed by a 4-h post-test sampling and questionnaire period. Testing for each participant began at 11:00am and ended by 4:15 pm. Blood was drawn at four different points during this time period (30 min before the TSST and 30, 90, and 240 min post-TSST). Participants were given specific instructions to refrain from excessive physical activity on the day of the testing, consuming alcohol for 12 h before their arrival, and eating and drinking (besides water) for 2 h prior to the testing session. The TSST was scheduled to begin at 12:00 pm to minimize the effects of circadian changes in cortisol. Detailed information on study procedures, including details on the TSST, has been reported previously [63].

The original motivation for this data collection was to examine differences in gene expression due to ELA status. For the purpose of the current investigation, we combined data from all participants, including those who had experienced ELA and controls. Exploratory analyses in Study 2 further examined differences in DNAm estimates of immune cell proportions by ELA status. ELA status of participants was assessed by a trained clinical interviewer during a phone interview using the Stressful Life Events Screening Questionnaire [64], as described previously [63]. BMI was obtained by collecting weight and height information from each participant and then calculating their standard weight to height-squared ratio. Ethnicity was self-reported as either “Hispanic” or “Non-Hispanic”.

Whole blood samples were repeatedly collected via an IV catheter into the antecubital vein. Blood samples collected in 4 mL EDTA tubes were sent to Quest Diagnostics for CBC analysis within 24 h. Blood samples collected in 10 mL EDTA tubes were immediately centrifuged for 10 min at 1500 g prior to collection of plasma. Peripheral blood mononuclear cells (PBMCs) were then isolated through density-gradient centrifugation using Ficoll. A small fraction of granulocytes in PBMC samples may have been retained (mean remaining granulocyte composition was 1.6% according to DNAm estimates) during processing. DNA was extracted from PBMCs using QIAmp mini kit (Qiagen) and sent to the Genome Sciences Core at The Pennsylvania State University for whole-genome DNAm analysis.

### DNAm quality control and immune cell proportion estimations

For both studies, DNA was bisulfite converted and processed by either McGill University (Study 1) or the Genome Sciences Core at The Pennsylvania State University (Study 2). DNAm levels were probed using the Illumina Infinium EPIC array [26]. EPIC array idat imaging files were converted to DNAm M and *β* value matrices with the *minfi* [65] package using R statistical software (R v4.1.2). All samples that had an average probe detection *p* value > 0.05 were excluded from our analyses. Sample normalization was performed according to recommendations by Salas and colleagues using the noob normalization method (which has been shown to decrease technical variation between batches [66]) in the *minfi* package [29] and DNAm estimates of blood cell proportions were computed using the *ProjectCellType_CP* function in the FlowSorted.Blood.EPIC package, which is equivalent to the *ProjectCellType* function in *minfi*. The “FlowSorted.BloodExtended.EPIC” library [29] was used as reference data for blood cell proportion estimates in both studies.

### Modeling CBC and DNAm estimates

The results from CBC tests and the DNAm estimates of immune cell proportions do not report the same categories of immune cell subtypes. CBC tests report neutrophils, lymphocytes, monocytes, eosinophils, and basophils whereas the recent Salas et al. DNAm estimates [29] provide a much larger subset of lymphocytes such as memory and naïve CD4T, CD8T, and B-cells, as well as natural killer (NK) cells and regulatory T-cells. All granulocytes (neutrophils, eosinophils, and basophils) were modeled in Study 1, whereas only PBMCs (composed only of monocytes and lymphocytes) were used in Study 2. The following formulae were used to compare the differing cell type reports from CBC and DNAm estimates:

$${CBC}_{Neutrophils}\leftrightarrow {DNAm}_{Neutrophils}$$

$${CBC}_{Lymphocytes}\leftrightarrow {DNAm}_{Naive B-Cells}+{{DNAm}_{Memory B-Cells}+{DNAm}_{Regulatory T-Cells}+DNAm}_{NK-Cell}+{DNAm}_{Naive CD4T}+{DNAm}_{Memory CD4T}+{DNAm}_{Naive CD8T}+{DNAm}_{Memory CD8T}$$

$${CBC}_{Monocytes}\leftrightarrow {DNAm}_{Monocytes}$$

$${CBC}_{Eosinophils}\leftrightarrow {DNAm}_{Eosinophils}$$

$${CBC}_{Basophils}\leftrightarrow {DNAm}_{Basophils}$$

### Statistical analysis

#### Study 1

In Study 1, Pearson correlation coefficients were calculated to test the association between CBC and DNAm estimates of immune cell proportions. Bland–Altman analyses [67, 68] were then performed to test for the concordance between each cell type’s CBC and DNAm estimates. Briefly, Bland–Altman analysis is a widely used statistical method to test the agreement between two types of measurement constructs, usually one of which is considered the “gold standard”. The mean of the two measurements is plotted on the x-axis and their difference is plotted on the y-axis. Usually, these analyses also include information on the mean difference between measurements and what are called the Limits of Agreement (95% confidence intervals on the mean difference between methods). (See [67, 68] For a more in-depth treatment of Bland–Altman analysis).

Multivariate linear regression was used to test whether the presence of CM significantly altered the concordance of CBC and DNAm estimates of immune cell proportions. The difference between CBC and DNAm estimates [DNAm estimate − CBC estimate] was treated as the outcome and CM was treated as a predictor. Additional covariates included age, sex, BMI, household income, average Tanner measurements for pubertal stage, race, and ethnicity. Although income was assessed as a categorical variable, it was treated as continuous. “White” and “non-Hispanic” racial and ethnic categorizations were treated as reference groups due to the fact that they were the largest groups in the sample. Continuous covariates were sample-mean centered and dichotomous covariates (0 or 1) were coded as − 0.5 or 0.5 to allow for easier interpretation of regression estimates for the sample mean.

#### Study 2

In Study 2, correlation coefficients were calculated to test the association between CBC and DNAm estimates of immune cell proportions. A repeated-measures Bland–Altman analysis [69] for each cell type’s CBC and DNAm estimates was used to test for the concordance of these values, as described in Study 1.

Due to the repeated-measures study design, a multilevel modeling framework was used to test whether acute psychosocial stress significantly altered the concordance of CBC and DNAm estimates of immune cell proportions. The difference between CBC and DNAm estimates of cell proportions [DNAm estimate − CBC estimate] was treated as the outcome and the time-point was treated as the predictor to determine if there was significant variation in difference scores across time-points. Individual random effects were included for the model intercept and additional covariates included sex, age, BMI, ethnicity, and ELA status. All variables, other than time-point, were mean centered as described for Study 1.

## Supplementary Information

**Additional file 1.** Supplementary Figures.

**Additional file 2.** Supplementary Tables.

## Data Availability

The datasets used and/or analyzed during the current study are available from the corresponding author on reasonable requests.

## Abbreviations

DNAm: DNA methylation
EWAS: Epigenome-wide association studies
CBC: Complete blood count
CpG: 5ʹ-cytosine-phosphate-guanine-3ʹ
CM: Child maltreatment
ELA: Early-life adversity
CHS: Child Health Study
TSST: Trier Social Stress Test
CWIS: Pennsylvania’s Statewide Child Welfare Information System
BMI: Body mass index
PBMCs: Peripheral blood mononuclear cells
NK: Natural killer cells
