# Supplementary material for: Investigating the effects of maltreatment and acute stress on the concordance of blood and DNA methylation methods of estimating immune cell proportions
Source: Clin Epigenetics. 2023 Feb 28;15:33. doi: 10.1186/s13148-023-01437-5 (PMC9976543; doi:10.1186/s13148-023-01437-5)
Supplement: Supplementary file 1 — Additional file 1. Supplementary Figures. [file 13148_2023_1437_MOESM1_ESM.docx]

**Supplementary Figures:**

Figure S1 – Study 1: Blood cell proportion distributions.

Figure S2 – Study 2: Blood cell proportion distributions.
